# How does Chronic Stress Impact Bioage and Inflammation among Older Black and White Adults?

**DOI:** 10.1093/geroni/igaf122.2018

**Published:** 2025-12-31

**Authors:** Lauren Brown, Patricia Louie, Calley Fisk, Cameron Brown, Kenya Luna

**Affiliations:** University of Southern California, Los Angeles, California, United States; University of Washington, Seattle, Washington, United States; University of Southern California, Los Angeles, California, United States; University of Southern California, Los Angeles, California, United States; University of Southern California, Los Angeles, California, United States

## Abstract

Prior research suggests that exposure to stressful events contributes to disparities in biological health in older adulthood. Yet, to understand the extent to which Black older adults bear a disproportionate stress and health burden, we consider Black-white differences in not only stress exposure, but also stress appraisal—how upsetting the exposures are perceived to be across five domains (health, financial, residential, relationship and caregiving). Data come from 4,912 adults ages 56+ from the 2016 Health and Retirement Venus Blood Study. Black older adults are more exposed to chronic stressors than white older adults and are more likely to rate health, financial, and housing stressors as upsetting. White older adults are more likely to rate relationship and caregiving stressors as upsetting. Stratified models show greater exposure to stressors and appraising stressors as upsetting are associated with increased bioage and inflammation for White older adults. For Black older adults, exposure and appraisal was not predictive of bioage or inflammation. Domain specific analyses show exposure to health, financial and housing strain drives the association between stress, bioage and inflammation among White older adults. For Black older adults only health problems are associated with increased bioage and inflammation. Findings suggest exposure and appraisal have varying consequences for biological health. Black older adults continue to demonstrate remarkable fortitude in the face of chronic stressors. Alternatively, our data may not capture the stress process for Black older adults, suggesting we need to recalibrate our measures to include culturally relevant approaches to measuring stress and bioage.

